# Early rupture of a tetrafluoroethylene loop due to fixation with polypropylene resulting in recurrent mitral valve regurgitation; a word of caution

**DOI:** 10.1093/jscr/rjaf393

**Published:** 2025-06-08

**Authors:** Keijiro Mitsube, Naohiro Wakabayashi, Masahiro Tsutsui, Ryohei Ushioda, Hiroyuki Kamiya, Tsutomu Fujita

**Affiliations:** Department of Cardiovascular Surgery, Sapporo Cardiovascular Clinic, 1-1-30 Kita 49-jo Higashi, Higashi-ku, Sapporo, Hokkaido,007-0849, Japan; Department of Cardiovascular Surgery, Sapporo Cardiovascular Clinic, 1-1-30 Kita 49-jo Higashi, Higashi-ku, Sapporo, Hokkaido,007-0849, Japan; Department of Cardiac Surgery, Asahikawa Medical University, 2-1-1-1 Midorigaoka Higashi, Asahikawa, Hokkaido 078-8510, Japan; Department of Cardiac Surgery, Asahikawa Medical University, 2-1-1-1 Midorigaoka Higashi, Asahikawa, Hokkaido 078-8510, Japan; Department of Cardiac Surgery, Asahikawa Medical University, 2-1-1-1 Midorigaoka Higashi, Asahikawa, Hokkaido 078-8510, Japan; Department of Cardiology, Sapporo Cardiovascular Clinic, 1-1-30 Kita 49-jo Higashi, Higashi-ku, Sapporo, Hokkaido, 007-0849, Japan

**Keywords:** mitral valve repair, mitral valve regurgitation, ePTFE loop, suture material

## Abstract

This case report describes early rupture of an expanded polytetrafluoroethylene (ePTFE) loop used in mitral valve repair, likely due to leaflet fixation with 5/0 polypropylene suture. A 52-year-old man developed recurrent mitral valve regurgitation 3 weeks postoperatively. Reoperation revealed rupture at the fixation site. This case suggests that ePTFE may be a more suitable material than polypropylene for leaflet fixation to prevent early loop failure.

## Introduction

Mitral valve repair using premeasured expanded polytetrafluoroethylene (ePTFE) loops to treat leaflet prolapse is widely adopted and recognized as a standard procedure [1, 2]. However, there is no consensus regarding the optimal suture material for fixing the loops to the leaflet. In this case, we report the early rupture of an ePTFE loop fixed with 5/0 polypropylene sutures, which led to the recurrence of mitral valve regurgitation (MR). We discuss the potential advantages of using ePTFE as the suture material for loop fixation to prevent early failure.

## Case report

**Figure 1:**
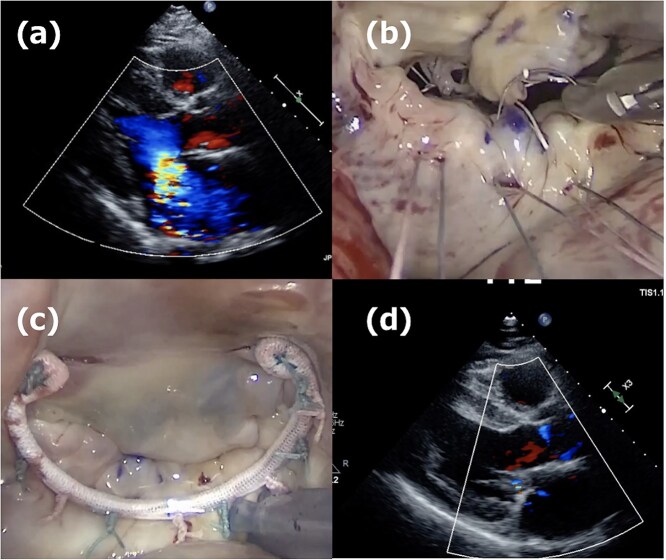
First operation images. (a) Severe mitral regurgitation caused by A2 prolapse. (b) Intraoperative image showing the placement of four 22-mm ePTFE loops fixed to the A2 segment with 5/0 polypropylene sutures. (c) The 36-mm CG-future band (Medtronics) was applied for mitral annuloplasty. (d) One week postoperative echocardiogram showing no residual mitral regurgitation.

**Figure 2:**
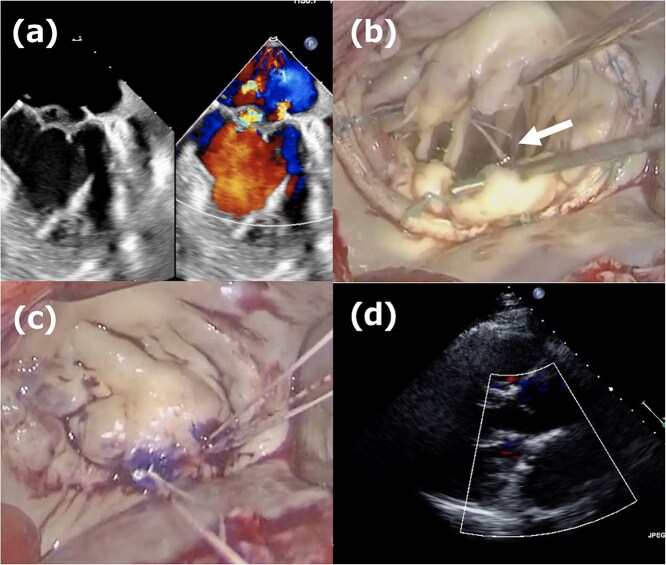
Second operation images. (a) Moderate mitral regurgitation caused by partial prolapse of the A2 segment, observed 3 weeks after the initial operation. (b) Intraoperative image showing the rupture of the ePTFE loop at the fixation site onto the leaflet during reoperation. (c) Three 20-mm long CV4 loops were attached to the posterior papillary muscle, and those loops were fixed to the A2 segment with CV4. (d) One week postoperative echocardiogram showing no residual mitral regurgitation after reoperation.

A 52-year-old man presented with chest discomfort on exertion. Transthoracic echocardiography (TTE) revealed severe MR due to a large prolapse of the A2 segment (Fig. 1a). Minimally invasive mitral valve repair was performed through right-sided small thoracotomy. The prolapsed A2 segment was treated with four 22-mm-ePTFE loops (CV4, Gore-Tex, W.L. Gore & Associates, Flagstaff, AZ) attached to the anterior papillary muscle. These loops were fixed to the A2 segment with 5/0 polypropylene sutures (Prolene, Ethicon, NJ) as shown in Fig. 1b. For annuloplasty, a 36-mm CG-Future band (Medtronic, MN) was applied (Fig. 1c). Intraoperative transesophageal echocardiography (TEE) and postoperative TTE showed no residual MR (Fig. 1d), and the patient was discharged on postoperative day 8. At a routine outpatient follow-up clinic 3 weeks later, a severe systolic murmur was detected, and TEE revealed a moderate MR. Partial prolapse of the A2 segment, which was treated at the first operation using the loop technique, was observed (Fig. 2a), and the lactate dehydrogenase level was 1599 U/L, indicating hemolysis. Although the patient remained asymptomatic, reoperation was recommended due to his relatively young age.

The second operation was performed 1 month after the initial operation. The approach employed was identical to that of the first operation; right-sided minithoracotomy was performed via the fourth intercostal space. Although access to the heart was more challenging due to adhesions, the operation proceeded in the usual manner, including femoral cannulations, aortic cross-clamping with antegrade cardioplegia, and right-sided left atriotomy. The most medial loop was ruptured at the fixation site onto the leaflet (Fig. 2b). The previously implanted annuloplasty band was removed, and three 20-mm CV4 loops were attached to the posterior papillary muscle and fixed to the A2 segment with CV4 (Fig. 2c). Thereafter, a new 34-mm annuloplasty ring (Physio II, Edwards Lifesciences, CA) was then implanted. Postoperative TTE confirmed no residual MR (Fig. 2d), and the patient remained free of recurrence at 1-year follow-up.

## Discussion

Mohr *et al.* introduced the loop technique in 1999 for mitral valve repair [3]. Due to its reproducibility, it has been widely adopted and became a standard procedure. In their original report, loops were made by CV5 sutures and anchored to the papillary muscle. Each loop was fixed onto the prolapsed leaflet with an additional CV5 suture. Until now, several modifications of the loop technique have been made [4]; however, no definitive consensus has been established on the suture material that should be used for fixing the loops onto the prolapsed leaflet. The Leipzig group used additional CV4 or CV5 sutures [3], whereas Shibata *et al.* used 5/0 polypropylene sutures and reported no cases of chordal rupture associated with these sutures [2]. Although rupture of CV4 or CV5 ePTFE neo-chordae is a rare complication, it is a recognized event that typically occurs in the late phase. Mutsuga *et al.* reported ePTFE rupture rates of 1.8% with CV5 and 0.2% with CV4, with median duration from the initial operation to reoperation of 99 and 78 months, respectively [5]. To our knowledge, this report is the first description of the early rupture of the ePTFE loop caused by polypropylene sutures for fixation onto the leaflet, suggesting that ePTFE may be an optimal suture material.
